# Computer-Aided Design (CAD) and Computer-Aided Manufacturing (CAM)-Based Fabrication of Andrews Bridge for Comprehensive Rehabilitation of Seibert Class III Ridge Defect: A Case Report

**DOI:** 10.7759/cureus.100067

**Published:** 2025-12-25

**Authors:** Deepesh Saxena, Tarannum Jindal

**Affiliations:** 1 Prosthodontics and Crown and Bridge, Subharti Dental College, Swami Vivekanand Subharti University, Meerut, IND

**Keywords:** alveolar ridge defect, andrews bridge, cad/cam, digital workflow, removable fixed partial denture

## Abstract

Alveolar ridge defects, especially Seibert Class III with combined horizontal and vertical loss, pose challenges in achieving optimal esthetics, function, and hygiene. The Andrews bridge, a hybrid fixed-removable prosthesis, offers an effective solution by combining the stability of a fixed component with the hygiene accessibility of a removable one. With advancements in digital dentistry, computer-aided design and manufacturing (CAD/CAM) now enable precise, fully digital fabrication of such prostheses. This case report presents a completely digital workflow for designing and fabricating an Andrews bridge, ensuring superior fit, esthetics, and functional harmony. The digital approach minimized laboratory errors, enhanced adaptation, and simplified clinical procedures. The final prosthesis provided improved facial support, phonetics, and hygiene maintenance. A fully digital Andrews bridge offers a predictable, efficient, and patient-centered solution for managing complex ridge defects where surgical or implant options are limited.

## Introduction

The alveolar bone is the part of the maxilla or mandible that contains the tooth roots, which are secured in place by the fibers of the periodontal ligament [1]. The term "localized alveolar ridge defect" refers to a confined volumetric deficiency of bone and soft tissue within the alveolar process. The management of an edentulous region with a ridge defect is a formidable challenge for a dentist. Multiple factors, including the quality and quantity of surrounding hard and soft tissues, the patient’s systemic health, and financial considerations, significantly influence treatment planning, clinical outcomes, and overall prognosis. Recent advancements in therapeutic modalities have facilitated the development of more personalized and predictable solutions for managing such cases. According to Siebert’s classification, which systematically categorizes ridge defects, and with the integration of computer-aided design (CAD) and computer-aided manufacturing (CAM) technology pioneered by Professor François Duret, it is now possible to fabricate highly precise and customized prostheses, such as Andrews bridges, thereby restoring both functional efficiency and aesthetic outcomes [2]. Andrews Bridge is a combination of fixed and removable prosthesis and is a propitious choice of treatment. Dr. James Andrews of the Institute of Cosmetic Dentistry, Amite, LA, USA, was the first to introduce this prosthesis in 1965 [3]. Although less frequently employed than conventional fixed prostheses or implant-supported restorations, the Andrews Bridge remains a valuable treatment option in select clinical circumstances. It is particularly indicated in cases of extensive alveolar ridge resorption, where conventional fixed prostheses cannot be satisfactorily utilized. The extent of its application varies across regions, with certain clinicians preferring it for its capacity to overcome specific anatomical limitations. However, its clinical application remains relatively limited, largely owing to the specificity of its indications and the broader accessibility of alternative treatment modalities, particularly implant-supported therapies [4]. This case report describes the rehabilitation of a Seibert Class III ridge defect using a digitally designed and milled Andrews bridge in a 44-year-old patient.

## Case presentation

A 44-year-old male patient reported to the Department of Prosthodontics and Crown & Bridge, Subharti Dental College and Hospital, Swami Vivekanand Subharti University, Meerut, India, with the chief complaint of an unaesthetic appearance resulting from missing teeth in the anterior maxillary region, which had been lost as a result of a road traffic accident.

**Figure 1:**
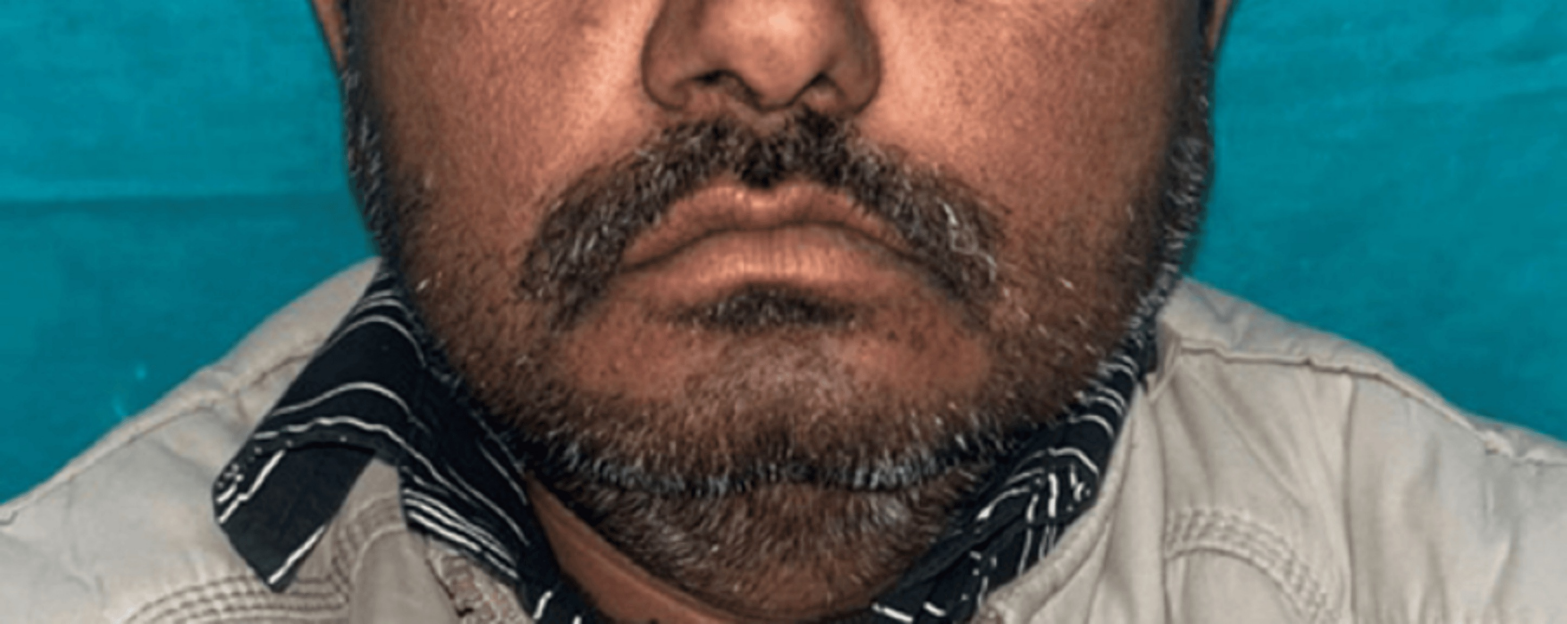
Frontal view of the patient Noticeable loss of upper lip support is evident, resulting in compromised facial aesthetics. Note: The patient consented to the use of their images in an open-access journal, and a written and signed consent statement from the patient was provided to the journal.

On extraoral examination, the patient presented with an overall symmetrical face; however, noticeable loss of upper lip support was evident, resulting in compromised facial aesthetics (Figure 1).

**Figure 2:**
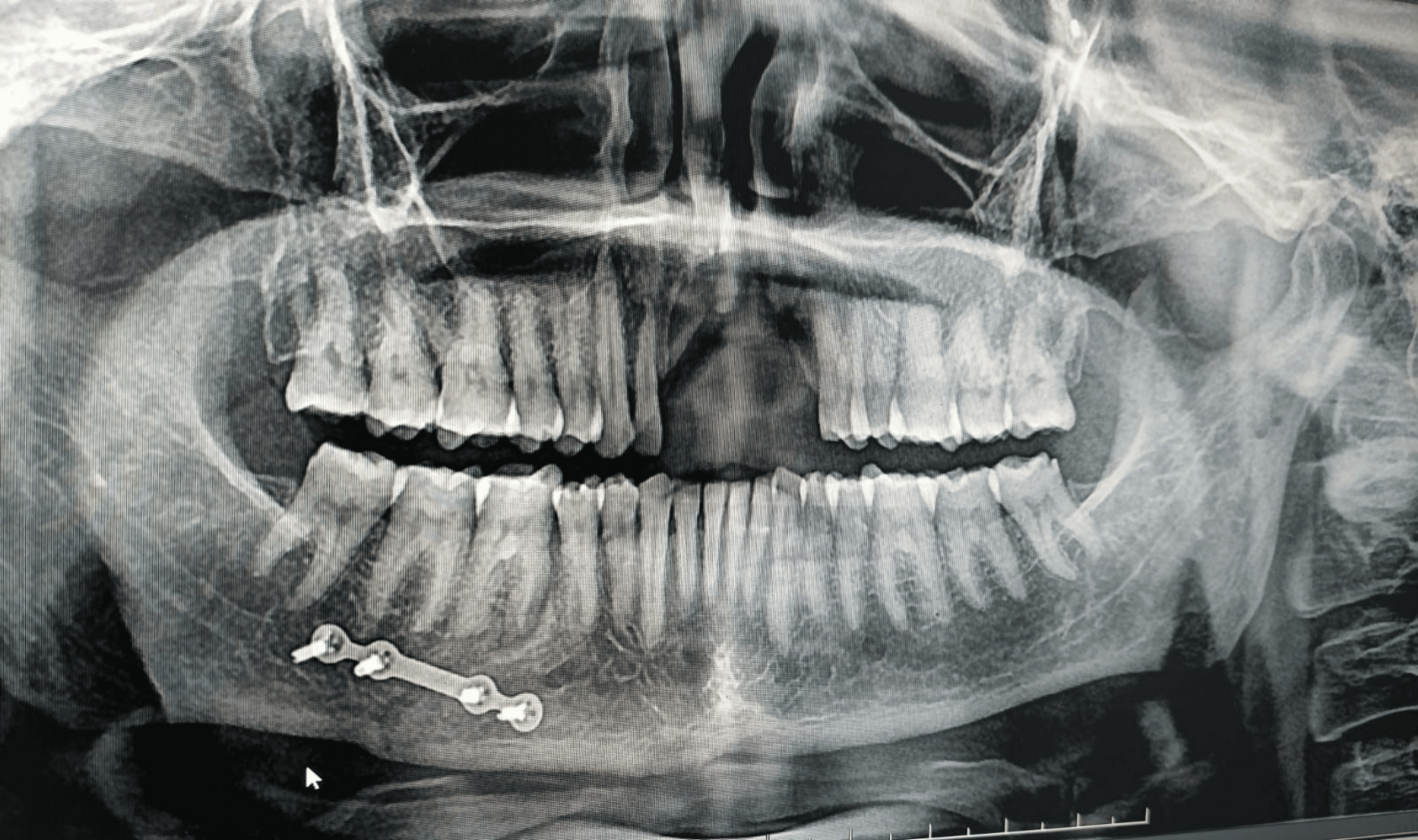
Panoramic radiograph (orthopantomogram) showing a well-aligned titanium fixation plate with four screws in the left mandibular body region.

No other gross facial asymmetry or scarring related to the previous trauma was observed. A panoramic radiograph (orthopantomogram) revealed a well-aligned titanium fixation plate with four screws in the left mandibular body region, consistent with prior open reduction and internal fixation of a mandibular fracture (Figure 2). 

**Figure 3:**
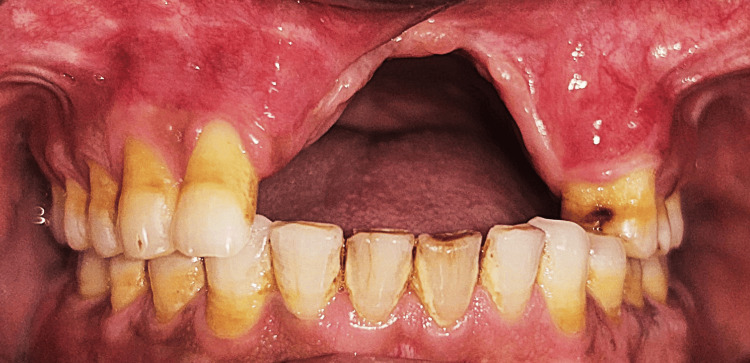
Intraoral view showing missing teeth 12, 11, and 21 with a vertical alveolar bone defect in the maxillary anterior region.

**Figure 4:**
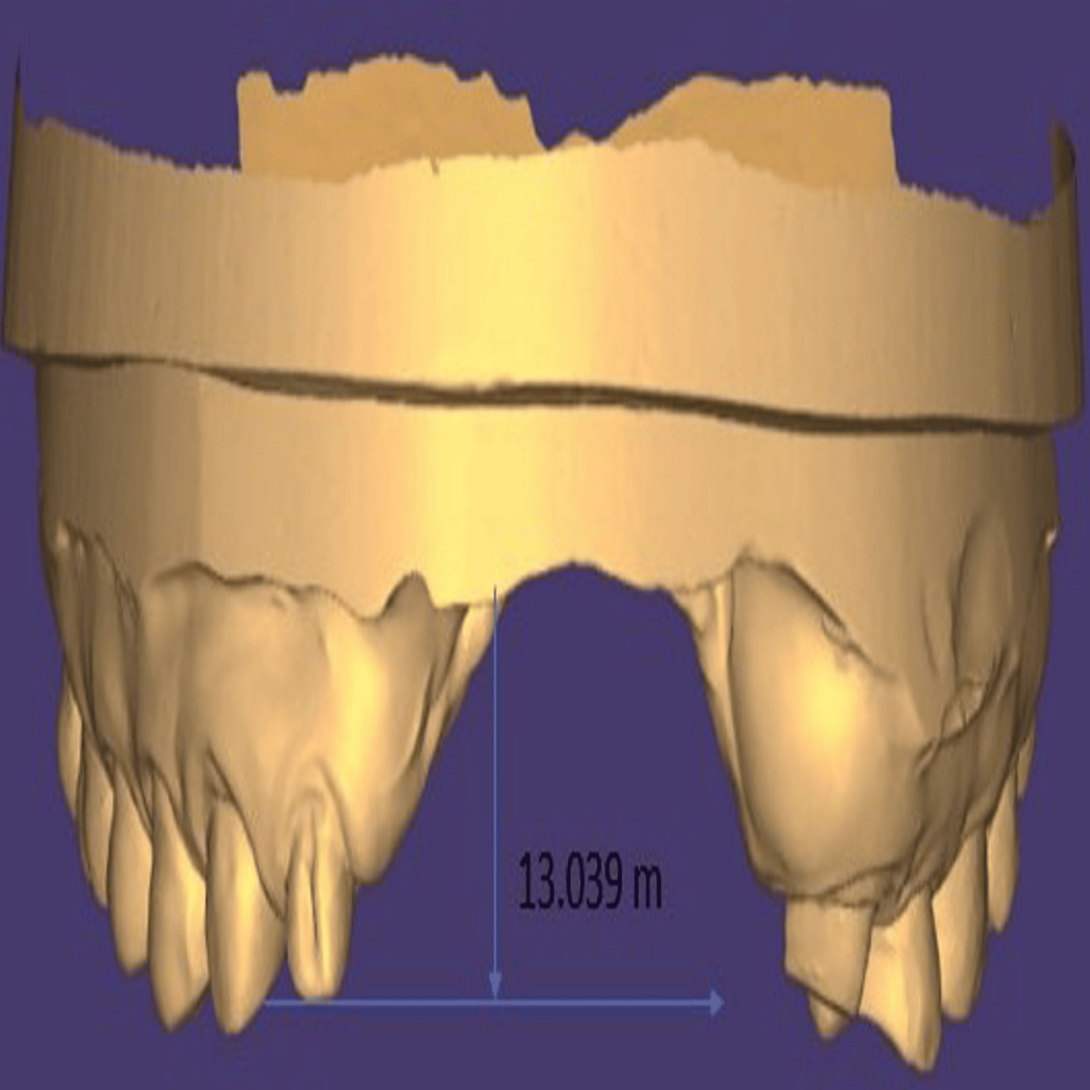
Vertical component of the defect Blender 4.1 software (Blender Foundation, Amsterdam, Netherlands) was used to demonstrate a 13 mm vertical component of the alveolar defect.

Dentition was largely intact with the absence of third molars, and no additional maxillofacial pathology was noted. Intraoral examination demonstrated the absence of teeth 11, 21, 22, and 23, accompanied by an alveolar bone defect present in the maxillary anterior region (Figure 3) with a vertical component measuring 13 mm as assessed using Blender 4.1 software (Blender Foundation, Amsterdam, Netherlands; Figure 4)

**Figure 5:**
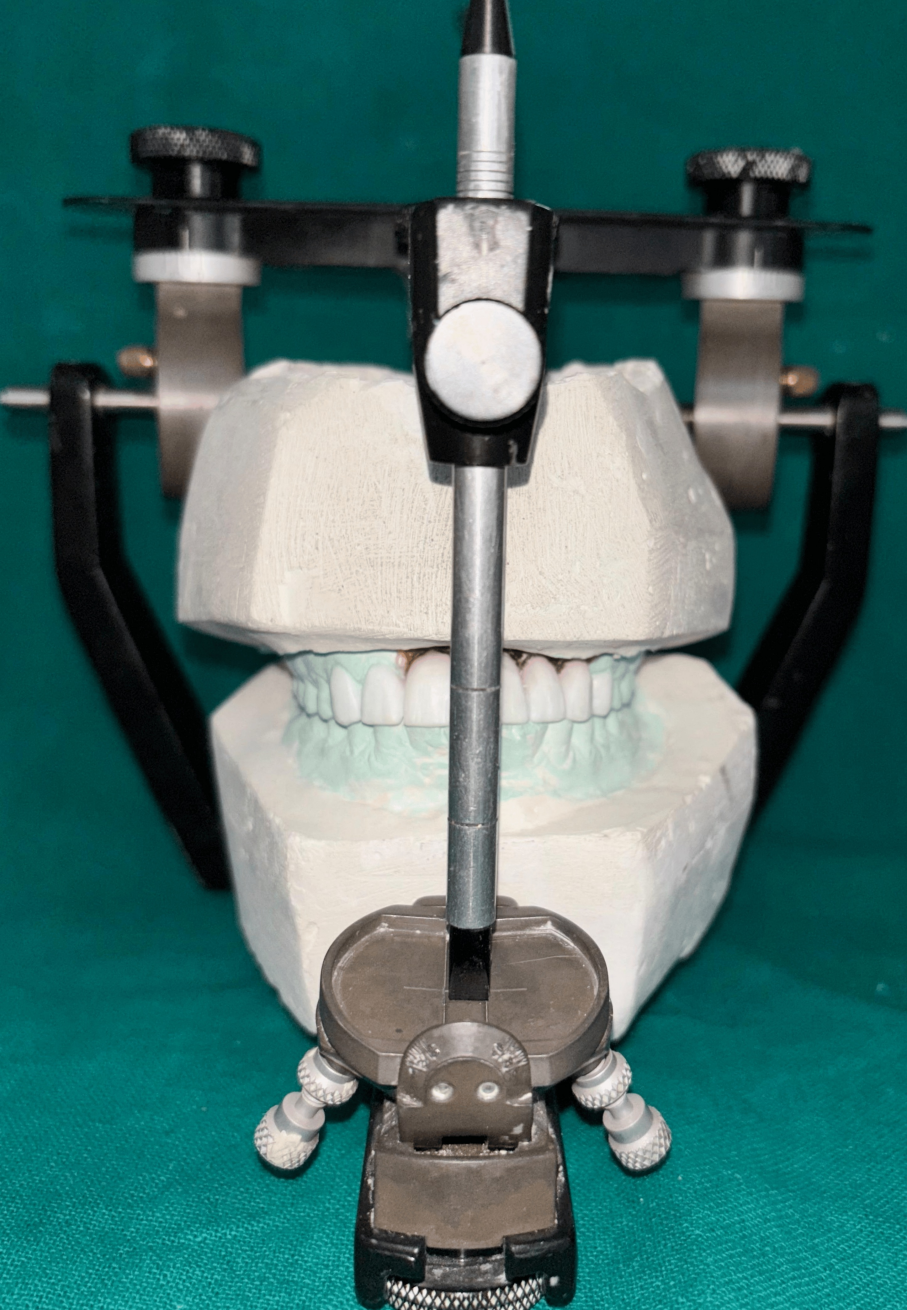
Diagnostic mock-up created with mock-up wax prior to tooth preparation.

Following the acquisition of diagnostic impressions, procedures to record the maxillomandibular relationship were undertaken. The maxillary cast was oriented using a Hanau springbow facebow (Whip Mix Corporation, Louisville, KY, USA) and subsequently transferred to a Hanau Wide-Vue semi-adjustable articulator (Whip Mix Corporation) to ensure accurate replication of the patient’s craniofacial relationships. Subsequently, a diagnostic mock-up was performed to simulate the anticipated esthetic and functional outcome (Figure 5).

**Figure 6:**
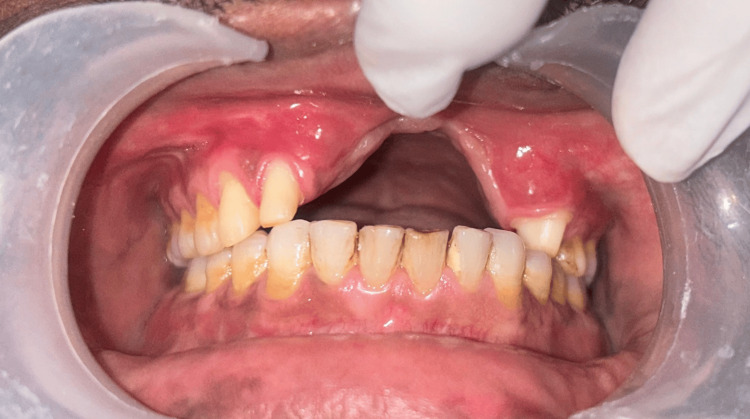
Teeth 12, 13, and 24 prepared for porcelain-fused-to-metal crowns forming the fixed component of the Andrews bridge system.

This procedure not only facilitated visualization of the proposed prosthetic rehabilitation for the patient but also served as a provisional guide to temporize the dentition following tooth preparation. Tooth preparation was carried out for teeth 12, 13, and 24, which were designated to receive metal-ceramic crowns and serve as abutments for the fixed component of the Andrews bridge (Figure 6).

**Figure 7:**
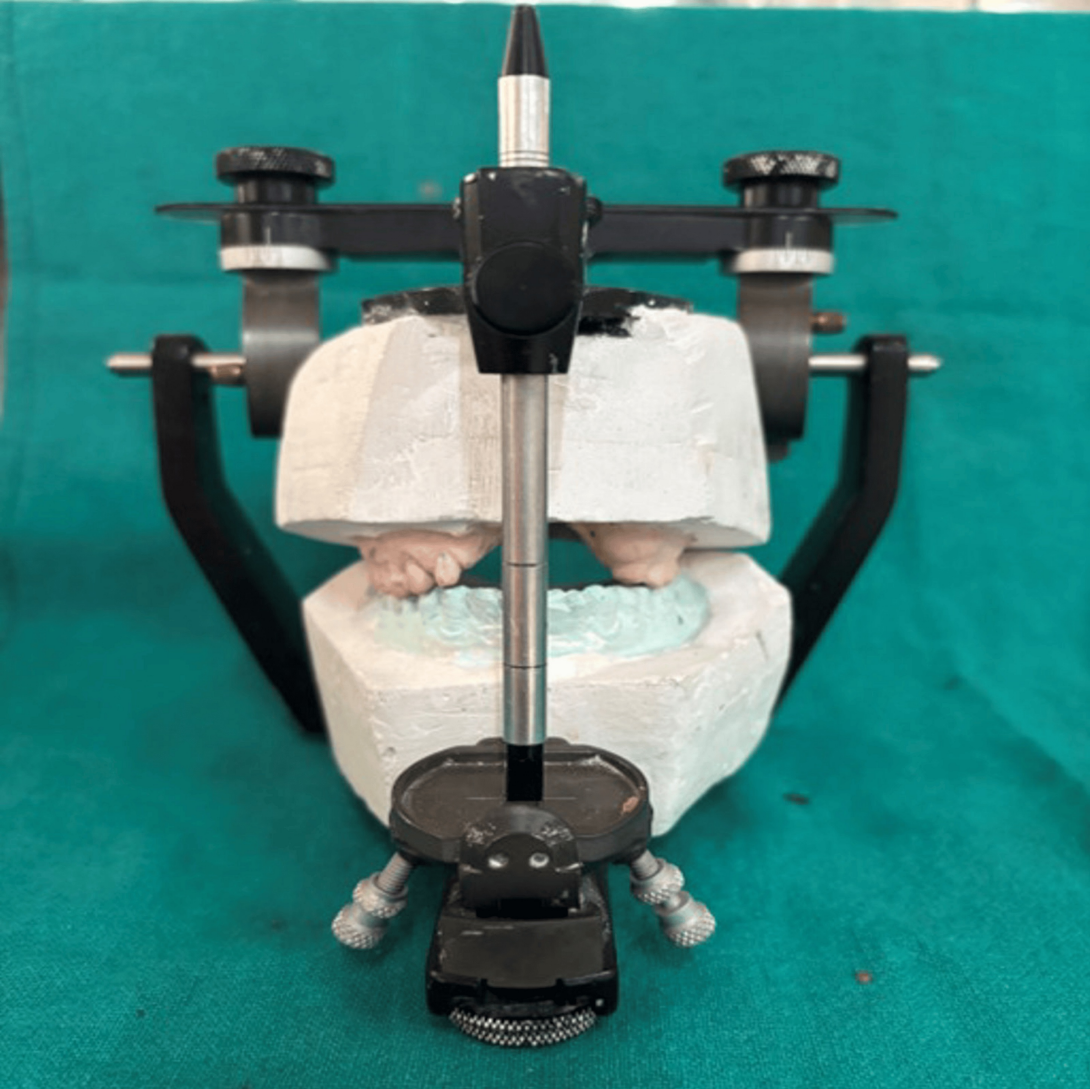
Definitive casts with prepared abutments mounted on a semi-adjustable articulator.

**Figure 8:**
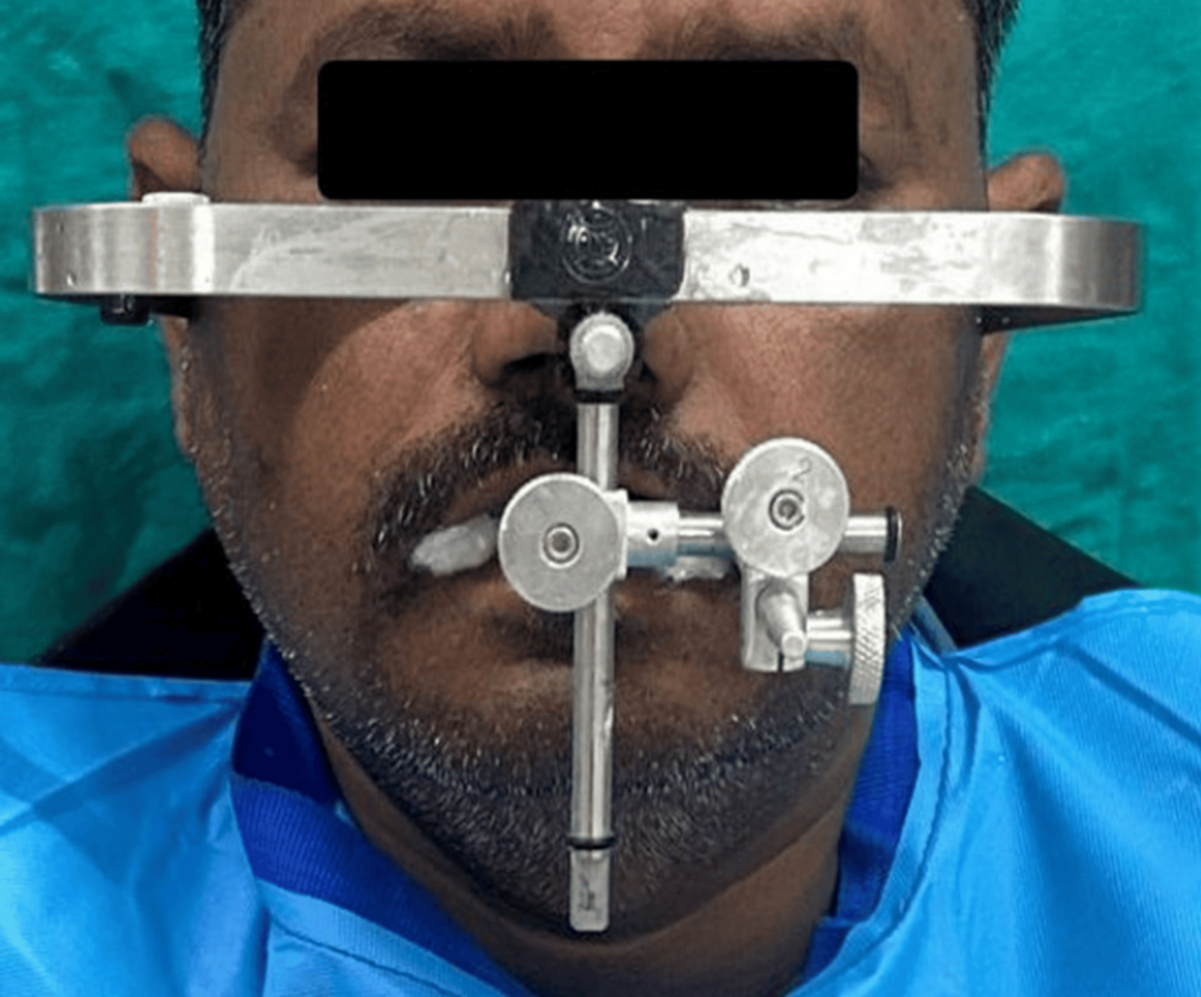
Orientation jaw relation was recorded using a Hanau Spring-Bow facebow (Whip Mix Corporation, Louisville, KY, USA). Note: The patient consented to the use of their images in an open-access journal, and a written and signed consent statement from the patient was provided to the journal.

The facebow record was repeated and subsequently transferred to a semi-adjustable articulator to ensure accurate maxillomandibular relationship (Figures 7, 8)

**Figure 9:**
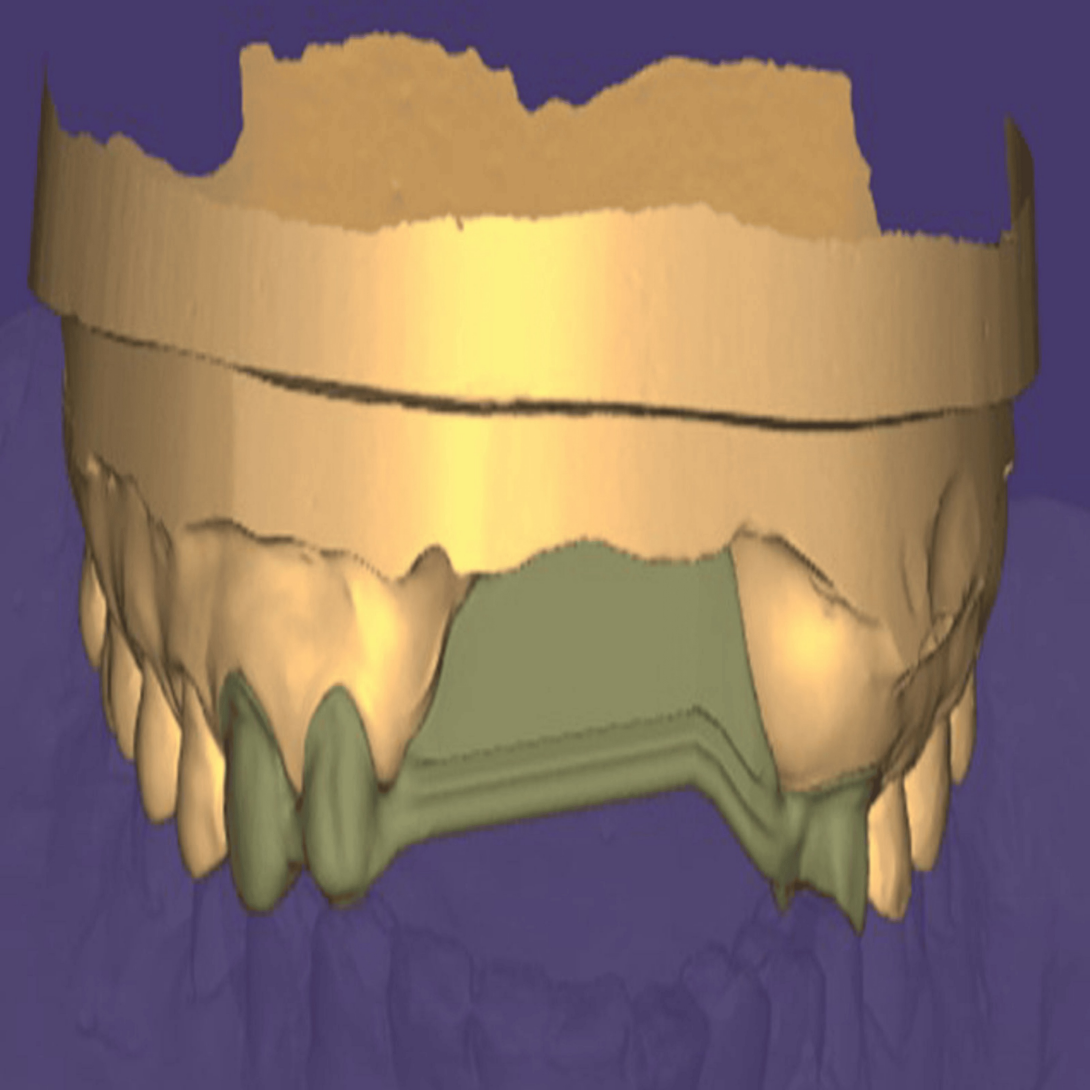
Frontal view of the computer-aided design-designed fixed prosthetic component created using Exocad software (Exocad GmbH, Darmstadt, Germany).

**Figure 10:**
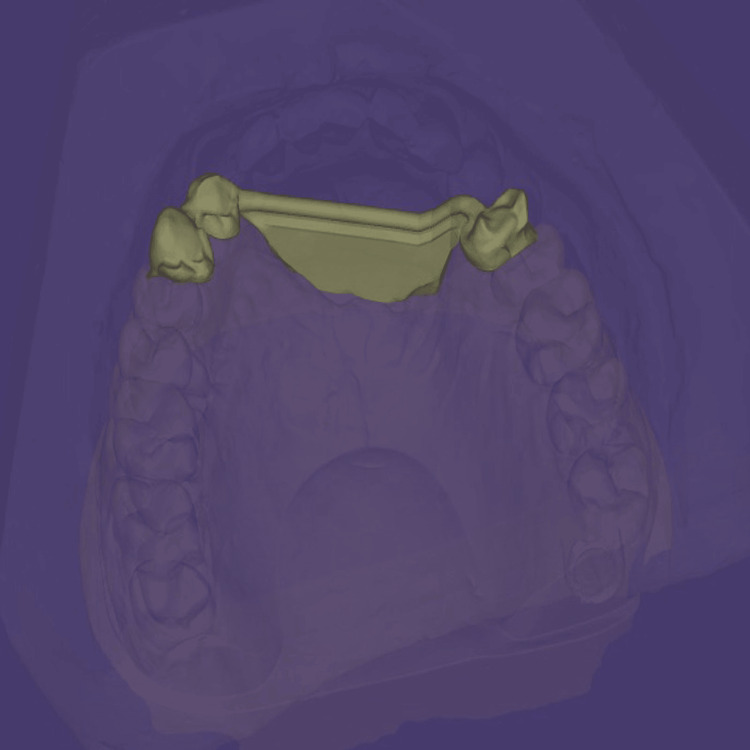
Palatal view of the computer-aided design-designed fixed prosthetic component created using Exocad software (Exocad GmbH, Darmstadt, Germany).

The definitive casts were digitized using an extraoral scanner, following which CAD was performed for the fixed component of the prosthesis. The design included the fabrication of metal copings and a Hader bar framework (Sterngold Dental LLC, Attleboro, MA, USA; Figures 9, 10).

**Figure 11:**
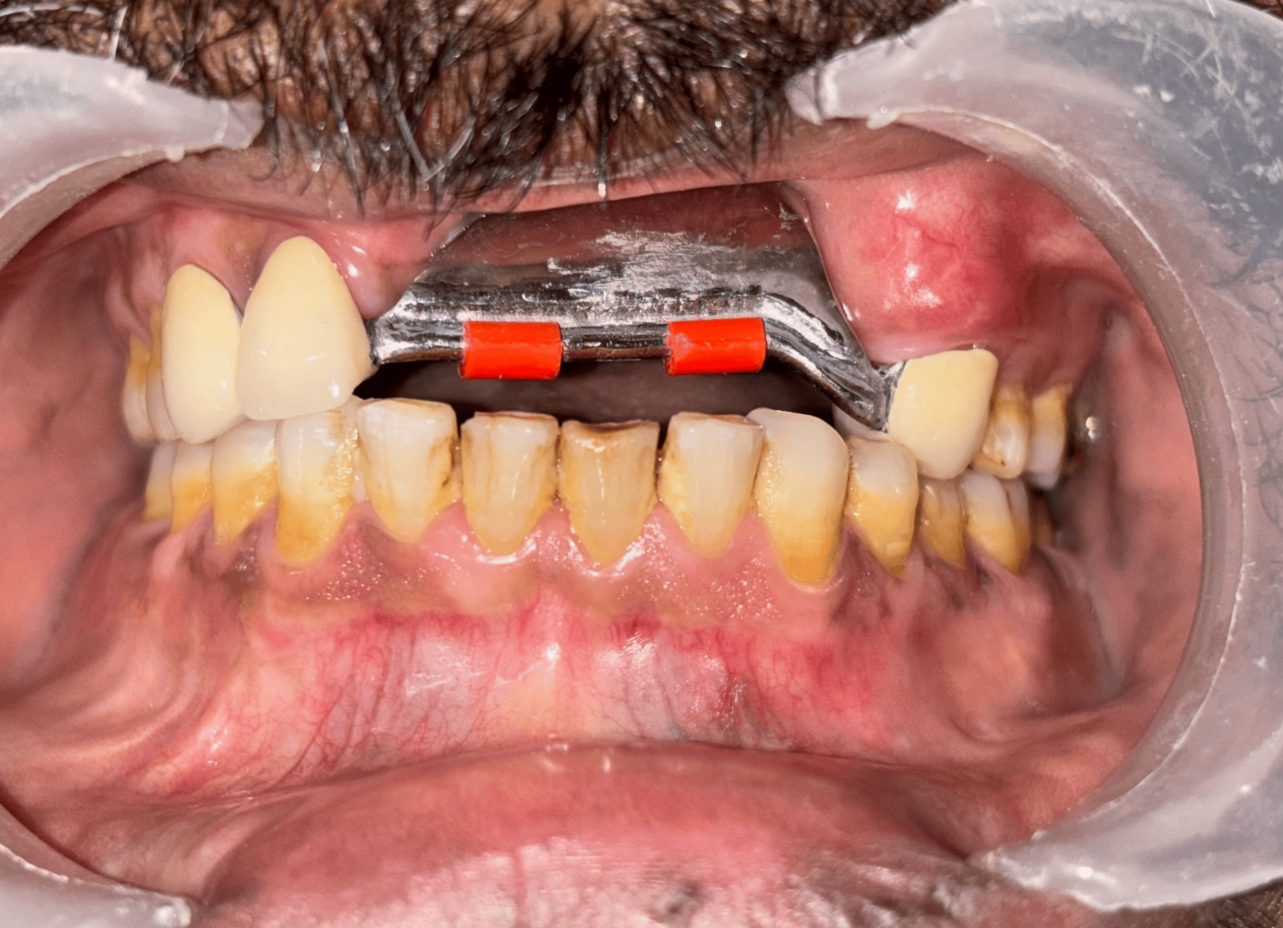
Try-in of the fixed prosthesis with the matrix part attached.

Subsequently, the prosthesis was fabricated using computer-assisted milling technology using Cobalt-Chromium material. The milled framework was evaluated intraorally to verify precision and marginal adaptation (Figure 11).

**Figure 12:**
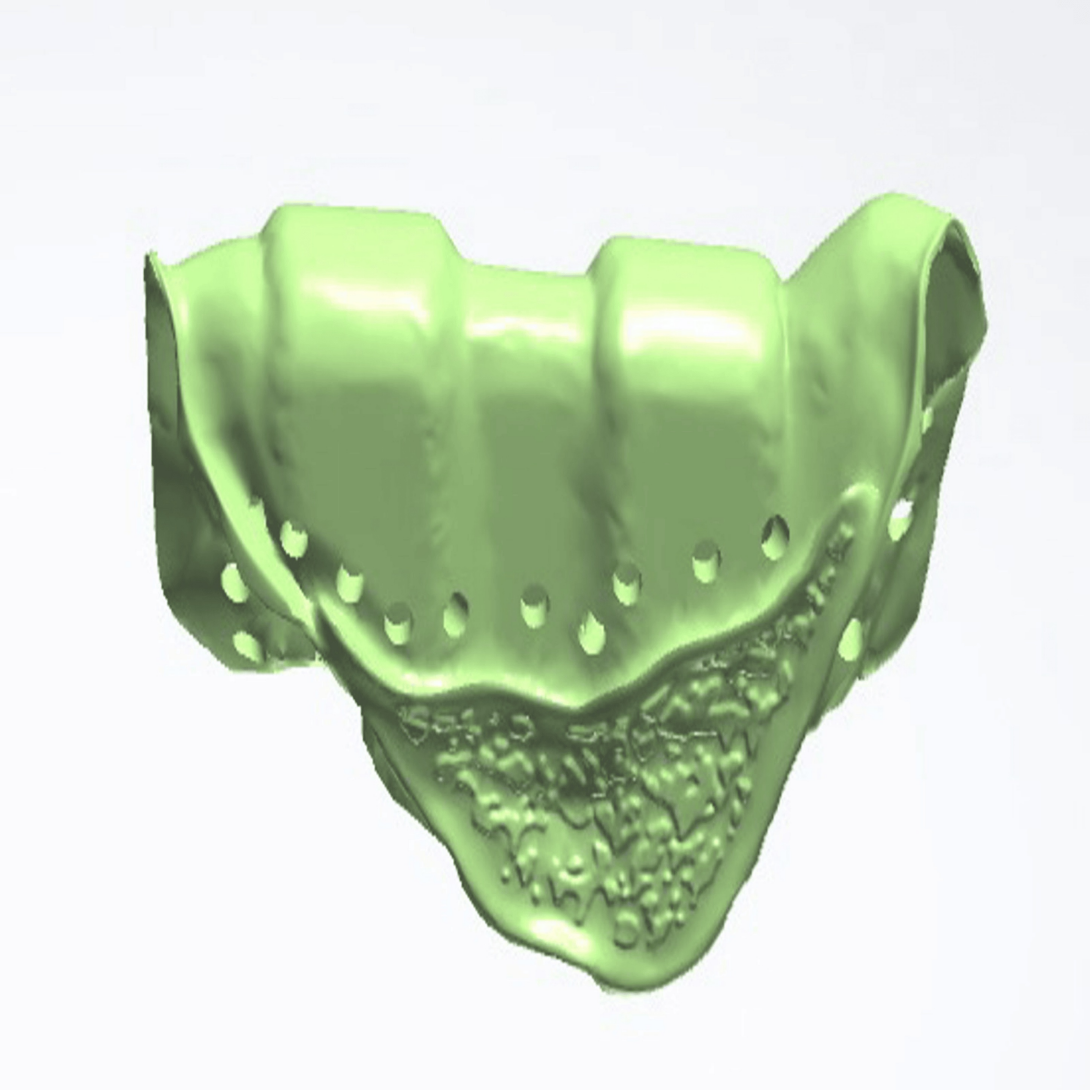
Frontal view of the digitally designed removable component of Andrews bridge system, created using Exocad software (Exocad GmbH, Darmstadt, Germany), demonstrating the precise adaptation and alignment of the prosthesis with the fixed portion.

**Figure 13:**
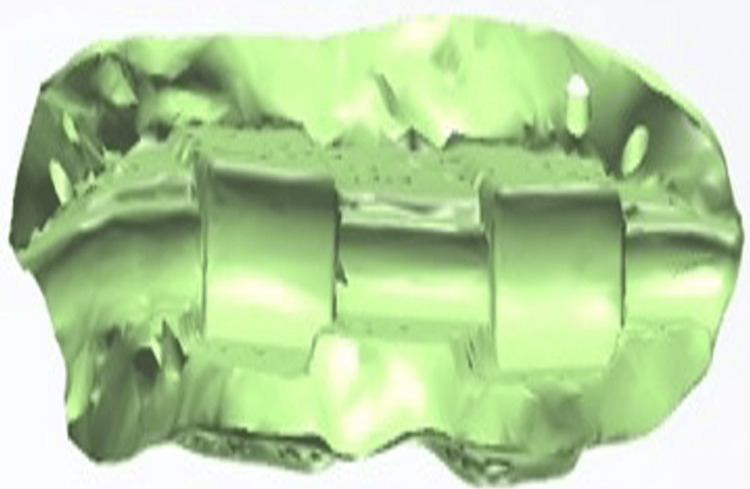
Internal surface of the removable prosthetic component of an Andrews Bridge system created using the Exocad software (Exocad GmbH, Darmstadt, Germany), illustrating the Hader bar (Sterngold Dental LLC, Attleboro, MA, USA) and incorporated nylon clip housings for mechanical retention.

Following confirmation of an accurate fit, porcelain layering was carried out to achieve the desired esthetics and function. After fabrication of the fixed portion, the cast model, along with the prosthesis, was scanned extraorally again, incorporating the Preci-Horix and Preci-Sagix female components for designing the removable cast partial denture (Figures 12, 13).

**Figure 14:**
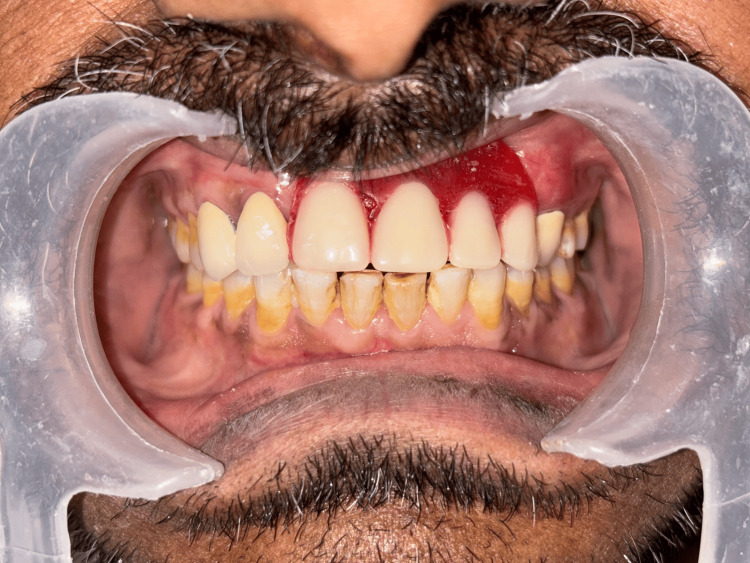
Try-in of the Andrews Bridge system performed to evaluate esthetics, phonetics, and anterior guidance.

The cast partial denture framework was milled from a cobalt-chromium alloy disk. Digital assessment of esthetic parameters was performed using Exocad software to ensure optimal functional and esthetic outcomes prior to finalization of the design. Teeth arrangement was carried out, followed by a clinical try-in to evaluate esthetics, phonetics, and anterior guidance (Figure 14).

**Figure 15:**
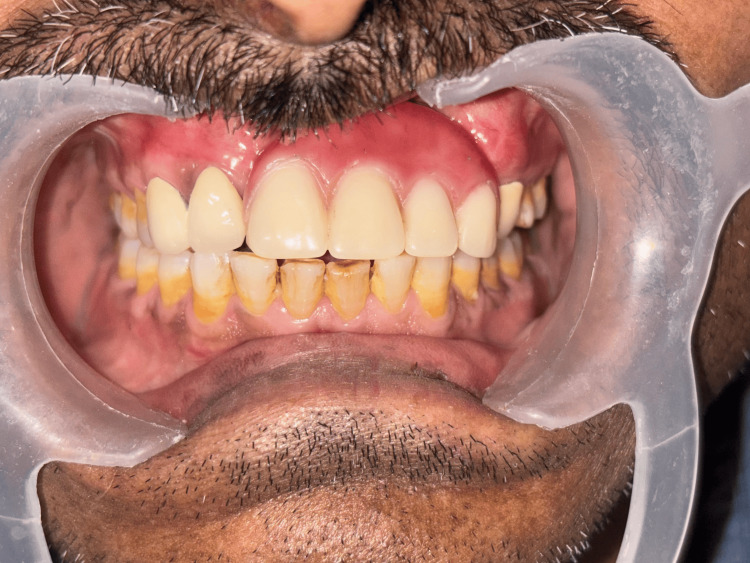
Insertion of the Andrews bridge system Cementation of the fixed prosthesis followed by insertion of the removable cast partial denture.

Upon satisfactory verification, acrylization of the removable component was performed. After completion of both the fixed and removable prostheses, indirect pick was done; the fixed component was cemented using glass ionomer luting cement, followed by insertion of the cast partial denture (Figure 15).

**Figure 16:**
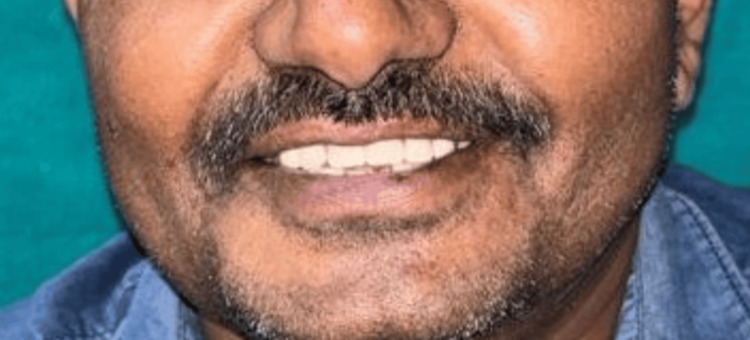
Extraoral frontal view showing the inserted Andrews bridge system. Note: The patient consented to the use of their images in an open-access journal, and a written and signed consent statement from the patient was provided to the journal.

The patient expressed satisfaction with the esthetic and phonetic outcomes. Post-insertion follow-up evaluations were conducted at one week, one month, six months, and one year, during which the prostheses demonstrated satisfactory function and patient compliance (Figure 16).

## Discussion

It has been observed that anterior tooth loss is commonly accompanied by alveolar bone resorption, with only about 9% of patients exhibiting no ridge defect. To assess the extent of ridge deformities in terms of form, function, and esthetics, Siebert (1983) proposed a classification system [5].

This system categorizes ridge defects into three classes based on the nature and extent of tissue loss: Class I: loss of tissue in the buccolingual direction; Class II: loss of tissue in the apicocoronal direction; Class III: combined loss of tissue in both buccolingual and apicocoronal directions.

The management of ridge deficiencies largely depends on their severity and morphological characteristics. Traditional surgical techniques such as cortico-cancellous onlay block grafting, distraction osteogenesis, guided bone regeneration, and ridge expansion are commonly employed, followed by appropriate prosthetic rehabilitation. However, in instances of extensive anterior ridge loss where surgical augmentation alone may not adequately restore form and function, the Andrews fixed-removable prosthetic system offers a reliable alternative. The digital methodology adopted in this case, which incorporated cast scanning and CAD/CAM technology, aligns with the contemporary shift in prosthodontics toward the integration of comprehensive digital workflows [6].

Direct metal laser sintering (DMLS) represents a transformative additive manufacturing technique that has attracted considerable attention across multiple research domains. Within prosthodontics and dental research, DMLS has been extensively investigated for its capacity to fabricate metal components with high precision and complex geometries. Numerous studies have evaluated the dimensional accuracy and mechanical performance of prostheses produced through DMLS, emphasizing its potential to deliver superior fit and functional reliability.

Prabhu et al. (2016) specifically examined the efficacy of DMLS in the fabrication of implant-supported frameworks, demonstrating its effectiveness in optimizing the manufacturing workflow while maintaining consistent quality standards. The ongoing advancements in DMLS research highlight its pivotal role in redefining prosthodontic practices by enabling enhanced customization, precision, and efficiency in the fabrication of dental restorations [7].

The Andrews bridge, a prosthetic system recognized for its distinctive design, has been widely examined in the literature for its clinical advantages. Gopi and Sahoo (2016) emphasized the superior esthetic outcomes achieved with the Andrews bridge, attributing these to the ridge-replacing acrylic removable segment [6]. In addition to esthetics, this component has been shown to improve phonetics and provide essential support to the facial musculature, as reported by Bhapkar et al. (2015) [8]. A notable characteristic of the Andrews bridge is the incorporation of a retentive latch mechanism for the removable segment, which contributes significantly to its stability and functional performance. Collectively, these findings highlight the Andrews bridge as a prosthodontic option that ensures enhanced esthetics, improved phonetics, and reliable facial support, thereby reinforcing its clinical value in comprehensive oral rehabilitation.

Despite these benefits, the system is not without limitations. The clinical and laboratory procedures are technique-sensitive, and food entrapment around the flange region may provoke tissue hyperplasia at the bar-ridge interface. Prosthesis failure may also result from inadequate soldering. Importantly, the Andrews bridge is contraindicated in patients with periodontally compromised abutments, and a minimum occluso-gingival height of 3-4 mm is essential for its functional success.

From a biomechanical perspective, the system derives its stability and retention entirely from the abutment teeth, directing occlusal forces along their long axes and thereby reducing harmful lateral stresses. The anatomical shaping of the pontic assembly enhances comfort and speech while also offering resistance to functional torque forces. A notable advantage of Andrews system is the removability of the pontic assembly, which facilitates oral hygiene maintenance and allows for adjustments as ridge resorption progresses.

## Conclusions

The present case report highlights the successful rehabilitation of a Seibert’s Class III ridge defect using a digitally designed and milled Andrews bridge. The integration of CAD/CAM technology and digital workflows facilitated enhanced precision, esthetics, and functionality, thereby addressing both clinical and patient-centered needs. The Andrews bridge continues to remain a valuable treatment modality in selected clinical scenarios, particularly in cases of severe ridge resorption where conventional fixed prostheses or implant-supported restorations may not be feasible. While its application is limited by technique sensitivity and case-specific contraindications, its unique combination of fixed and removable components offers significant advantages in terms of esthetics, phonetics, hygiene maintenance, and patient satisfaction. The incorporation of advanced digital manufacturing technologies, such as DMLS, further augments the accuracy, customization, and efficiency of the prosthetic outcome. Overall, the Andrews bridge, when supported by modern digital protocols, represents a viable and effective solution for managing complex ridge defects in contemporary prosthodontic practice.
